# Development of Safe and Effective RSV Vaccine by Modified CD4 Epitope in G Protein Core Fragment (Gcf)

**DOI:** 10.1371/journal.pone.0094269

**Published:** 2014-04-15

**Authors:** In Su Cheon, Byoung-Shik Shim, Sung-Moo Park, Youngjoo Choi, Ji Eun Jang, Dae Im Jung, Jae-Ouk Kim, Jun Chang, Cheol-Heui Yun, Man Ki Song

**Affiliations:** 1 Laboratory Sciences Division, International Vaccine Institute, Seoul, Republic of Korea; 2 Department of Agricultural Biotechnology and Research Institute for Agriculture and Life Sciences, Seoul National University, Seoul, Republic of Korea; 3 World Class University Biomodulation Major and Center for Food and Bioconvergence, Seoul National University, Seoul, Republic of Korea; 4 Graduate School of Pharmaceutical Sciences, Ewha Womans University, Seoul, Republic of Korea; University of Iowa, United States of America

## Abstract

Respiratory syncytial virus (RSV) is a major cause of respiratory tract infection in infants and young children worldwide, but currently no safe and effective vaccine is available. The RSV G glycoprotein (RSVG), a major attachment protein, is an important target for the induction of protective immune responses during RSV infection. However, it has been thought that a CD4^+^ T cell epitope (a.a. 183–195) within RSVG is associated with pathogenic pulmonary eosinophilia. To develop safe and effective RSV vaccine using RSV G protein core fragment (Gcf), several Gcf variants resulting from modification to CD4^+^ T cell epitope were constructed. Mice were immunized with each variant Gcf, and the levels of RSV-specific serum IgG were measured. At day 4 post-challenge with RSV subtype A or B, lung viral titers and pulmonary eosinophilia were determined and changes in body weight were monitored. With wild type Gcf derived from RSV A2 (wtAGcf), although RSV A subtype-specific immune responses were induced, vaccine-enhanced disease characterized by excessive pulmonary eosinophil recruitment and body weight loss were evident, whereas wtGcf from RSV B1 (wtBGcf) induced RSV B subtype-specific immune responses without the signs of vaccine-enhanced disease. Mice immunized with Th-mGcf, a fusion protein consisting CD4^+^ T cell epitope from RSV F (F_51–66_) conjugated to mGcf that contains alanine substitutions at a.a. position 185 and 188, showed higher levels of RSV-specific IgG response than mice immunized with mGcf. Both wtAGcf and Th-mGcf provided complete protection against RSV A2 and partial protection against RSV B. Importantly, mice immunized with Th-mGcf did not develop vaccine-enhanced disease following RSV challenge. Immunization of Th-mGcf provided protection against RSV infection without the symptom of vaccine-enhanced disease. Our study provides a novel strategy to develop a safe and effective mucosal RSV vaccine by manipulating the CD4^+^ T cell epitope within RSV G protein.

## Introduction

Respiratory syncytial virus (RSV), consisting of A and B subtype, is a major causative agent of severe lower respiratory tract disease in infants, young children, and the elderly worldwide. Nevertheless, there is no safe and effective RSV vaccine licensed for human use. Although considerable efforts have been invested for the development of safe and effective RSV vaccines, none has been successful owing to the difficulties in achieving the proper balance of safety and efficacy. Formalin-inactivated RSV (FI-RSV) was the first RSV vaccine candidate introduced in the 1960s. However, FI-RSV caused enhanced respiratory disease hallmarked by pulmonary eosinophilia and predominant Th2 type cytokine response following subsequent RSV infection in individuals who received this vaccine [1], [2].

The G glycoprotein of RSV, a major attachment protein, is a potentially important target for the induction of neutralizing antibodies and protective antiviral immune response [3], [4]. For example, BBG2Na, a subunit vaccine candidate, has shown to elicit immune response in small and large animals and been evaluated in human clinical trials [5]–[7]. Moreover, studies evaluating BBG2Na in combination with different adjuvants and by different routes of administration have further confirmed the role for the RSV G protein in protection against RSV [5]–[7]. Further, a study on the serum reactivity to various RSVG epitopes using sera harvested from RSV A- and B-infected human subjects reported a significant increase in cross-subtype IgG response against the central conserved region of the RSVG [8], suggesting that the Abs specific to the central conserved region of the RSVG may be able to neutralize both A and B subtypes of RSV and provide heterosubtypic protection. As such, various RSVG-derived vaccine candidates including recombinant vaccinia virus expressing RSV G protein (rVVG) have been evaluated. However, mice vaccinated with rVVG developed enhanced lung disease accompanied by pulmonary eosinophilia following intranasal RSV infection [9]–[11]. Further studies have suggested the induction of Th2-biased CD4^+^ T cell response concomitant with the secretion of excess Th2 cytokines as the cause of rVVG immunization leading to the enhanced lung disease [9], [12], [13].

It is worth noting that symptoms of immunopathology caused by rVVG immunization, such as eosinophilia and Th2-biased responses, interestingly, were similar to those caused by FI-RSV immunization [12], [14], [15]. Importantly, studies have further identified RSVG-specific subset of CD4^+^ T cells expressing Vβ14 TCR as the culprit behind the pulmonary eosinophilia and exaggerated Th2 cytokine production [16], [17]. Furthermore, studies have reported that mice immunized with the KLH-conjugated peptide corresponding to RSV G_184–198_, which is the CD4^+^ T cell epitope within RSV G protein, induces severe pulmonary eosinophilia upon live RSV challenge strongly suggesting the involvement of amino acid (a.a.) residues 184–198 of RSVG to the RSVG-mediated immunopathology [18].

Increasing numbers of studies have reported that immunization via sublingual route induces both systemic and mucosal immune responses against various antigens [19]–[21]. In addition, we have shown previously that RSV G protein fragment (Gcf), encompassing the amino acid residues 131–230, when delivered sublingually, elicits Ag-specific immune response in mice [22]. Gcf immunization with CT, as a mucosal adjuvant, induced higher level of antibody than those in mice immunized with Gcf alone. However, Gcf vaccine elicited significant lung eosinophilia when it was co-immunized with CT meaning that it still has the potential to cause immunopathological symptoms when it is combined with an adjuvant such as CT [22].

Most studies of RSV G protein were performed with RSV A subtype, and there has been only a limited amount of studies on RSV B subtype and its G protein, despite both A and B subtype are co-circulated. Therefore, in the present study, we engineered various modified recombinant RSV Gcfs to circumvent the potential RSVG mediated immunopathology against RSV A and B subtype. These modified Gcfs were evaluated for vaccine-induced immune response and vaccine-enhanced disease such as eosinophilia in the airway and body weight loss. These various Gcf-derivatives demonstrated a promising potential as novel RSV vaccine candidates.

## Materials and Methods

### 2.1. Ethics Statement

All animal studies were approved by Institutional Animal Care and Use Committee (IACUC) at International Vaccine Institute (Approval No. 2011-008).

### 2.2. Virus Preparation

RSV A2 strain and B strain (CH18537 or HRSV-B isolate, KR/B/10–12) were amplified as previously described [22], [23]. Briefly, virus was propagated in HEp-2 cells (ATCC, Manassas, VA) grown in MEM (Life Technologies, Grand Island, NY, USA) and harvested at day 3 or 4 post-infection when extensive syncytia were observed in infected cells. Virus titer was determined by standard RSV plaque assay.

### 2.3. Construction of Plasmids Expressing Various Gcf Proteins

**Figure 1:**
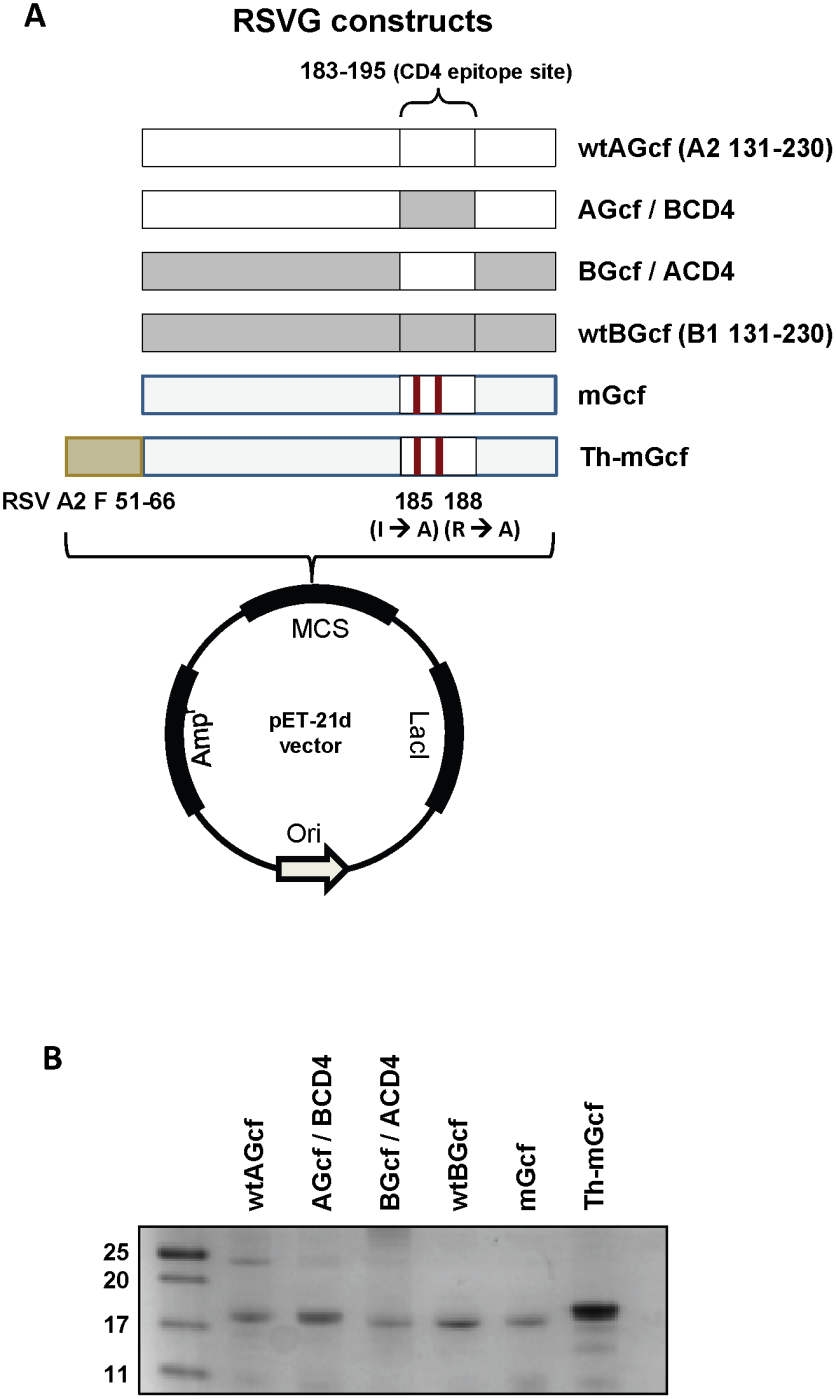
Construction of plasmids expressing various Gcf proteins and purified proteins. (A) wtAGcf, AGcf/BCD4, BGcf/ACD4, wtBGcf, mGcf or Th-mGcf genes were cloned into pET21d vector to express recombinant Gcf proteins in *E. coli* (B) The proteins expressed in E. coli were purified by His-tag affinity chromatography and separated by 15% SDS-PAGE.

Illustrative schemes of plasmid expressing each Gcf protein are depicted in Figure 1A. The plasmid containing wtAGcf gene was prepared as previously described [22]. The mGcf gene, which was designed to produce mutations at a.a. 185 and 188 inside the CD4^+^ T cell epitope region within wtAGcf, was generated with the forward primer (5′-TGG GCT GCC TGC AAA GCA ATA CCA AAC AAA AAA CCA GGA-3′) and the reverse primer (5′-GGG CCC AAG CTT GGG CTT GGT GGT GGG TAC TTC-3′) by site-directed mutagenesis using wtAGcf gene as the template. The Th-mGcf gene possessing RSV F protein CD4^+^ T cell epitope at N terminus of mGcf was generated with the primers 5′-CCC GAA TTC CC GGT TGG TAT ACC AGT GTT ATA ACT ATA GAA TTA AGT AAT ATT AAG GAA GTC AAG ACC AAA AAC ACA ACA-3′ and 5′-GGG CCC AAG CTT GGG CTT GGT GGT GGG TAC TTC-3′ by polymerase chain reaction (PCR) using plasmid pET21d-mGcf as the template. The wtBGcf (a.a. 131 to 230), AGcf/BCD4 and BGcf/ACD4 genes were synthesized by Bioneer (Korea). The CD4^+^ T cell epitope inside wtAGcf or wtBGcf (a.a. 183 to 195) was replaced with the corresponding region within wtBGcf (derived from RSV CH18537) or wtAGcf (derived from RSV A2), respectively, to create AGcf/BCD4 or BGcf/ACD4. The DNA sequences were confirmed by Macrogen (Seoul, Korea).

### 2.4. Expression and Purification of Various Gcf Proteins

*E. coli* BL21 (DE3) strain (Novagen) transformed with each plasmid was grown overnight at 37°C in Luria-Bertani (LB) medium supplemented with 100 µg/ml of ampicillin. The overnight culture was transferred into fresh LB medium and cultured at 37°C while shaking at 180 rpm until OD_600_ of 0.6∼0.8. Each protein expression was induced by adding IPTG of 0.5 M for 4 hrs and the cells were harvested by centrifugation at 6,000 rpm for 10 min. The cell pellets were suspended in binding buffer (20 mM Tris, 0.5 M Nacl, pH 7.9) and disrupted by sonication on ice. After sonication, the soluble and insoluble fractions were separated by centrifugation for 40 min at 20,000 rpm. For the Th-mGcf protein, the insoluble fraction was dissolved in binding buffer containing 6 M urea. After centrifugation for 30 min at 18,000 rpm, the supernatant was applied to a Talon metal affinity column (Clontech, Palo Alto, CA). For the wtA Gcf, wtB Gcf, AGcf/BCD4, B Gcf/ACD4 and mGcf, the soluble fractions were applied to a Talon metal affinity column. The columns were washed with binding buffer containing 20 mM imidazole, and then the proteins were eluted using an elution buffer (300 mM imidazole, 20 mM Tris, 0.5 M NaCl, pH 7.4). The purified proteins were dialyzed against 1 x PBS. The endotxoin in each purified protein was removed by using Triton X-114 as previously described [21]. The endotoxin level of each protein was measured by the limulus amebocyte lysate (LAL) assay kit according to the instructions (Lonza, Switzerland). To note, endotoxin levels of the proteins were less than 5 EU/mg. The purified proteins were electrophoresed on 15% SDS-PAGE and the bands were visualized by staining with Coomassie Brilliant Blue (Figure 1B). The protein concentration was determined by Bradford protein assay kit (Biorad, CA, USA). The purified proteins were stored at −80°C until use.

### 2.5. Mice and Immunization

Specific pathogen free, female BALB/c mice aged 6 weeks were purchased from Orient Bio Inc. (Korea) and all mice were maintained under specific pathogen-free conditions. Mice were immunized with 20 µg of each purified Gcf proteins with 2 µg of CT (List Biological Lab. Inc. Campbell, CA) via the sublingual (s.l.) route on day 0 and day 14. As control, mice were immunized with CT via sublingually, 1×10^5^ PFU of FI-RSV via foot-pad as described by Delgado et al. [24], or 1×10^5^ PFU of live RSV through intranasal (i.n.) route. For s.l. immunization, the anesthetized mice were immunized with 15 µl of prepared vaccines underneath the tongue using a pipette. Following s.l. immunization, mice were maintained with heads placed in ante flexion for 30 min. For i.n. immunization, total 20 µl of prepared vaccines were administered into each nostril of the anesthetized mice. Three weeks after the last immunization, the mice were challenged i.n. with 2×10^6^ PFU of live RSV A2 or 2×10^6^ or 4×10^6^ PFU of live CH18537 or KR/B/10–12 for B subtype RSV.

### 2.6. T Cell Response

Mice were immunized twice on days 0 and 14, and challenged with RSV A2 as described above. On day 4 post-challenge, the lungs were harvested and strained through 70-µm cell strainer (BD Biosciences, San Diego, CA, USA) using serum-free RPMI. To examine the cytokine-producing cells, lung mononuclear cells were stimulated with 10 µg/ml a.a. 183–195 G peptide (WAICKRIPNKKPG from wtAGcf, KSICKTIPSNKPK from wtBGcf) or 1 µg/ml of anti-CD3 and anti-CD28 antibodies (BD Biosciences) and incubated for 5 hr in the presence of Golgi Plug (BD Biosciences). The cells were then stained with anti-mouse CD4 (BD Biosciences) for surface markers. The cells were fixed, permeabilized using Cytofix/Cytoperm solution (BD Biosciences), and stained with anti-mouse IFN-γ. Stained cells were acquired using flow cytometry (FACS LSR II; BD Biosciences) and analyzed by FlowJo (Tree Star, San Carlos, CA, USA). For ELISPOT, the plates (Millipore, Billerica, MA, USA) were coated with 100 µl of coating anti-IFN-γ (e-Bioscience, San Diego, CA, USA) at a concentration of 10 µg/ml overnight at 4°C and blocked with RPMI-1640 (Life Technologies, Grand Island, NY, USA) containing 10% FBS (Lonza, Switzerland) for 30 min at 37°C. Then 2×10^5^ or 5×10^4^ lung cells were transferred into each well and stimulated with 10 µg/ml of G (183–195) peptide (WAICKRIPNKKPG) or F (51–66) peptide (MGWYTSVITIELSNIK) for 24 hr at 37°C. Following intensive washing with PBS, biotinylated anti-IFN-γ antibodies (e-Bioscience) were added into each well, and incubated overnight at 4°C. Next day, the plates were incubated with streptavidin-HRP (BD Biosciences) for 1hr at room temperature. Spots were developed by adding AEC-H_2_O_2_ chromogenic substrate (Sigma-Aldrich) and counted.

### 2.7. Measurement of Eosinophils in the BAL

BAL samples were collected as described previously [21] on day 4 post-challenge. Briefly, mice were sacrificed and BAL samples were collected using PBS via tracheotomy. Cells were separated from the BAL fluid by centrifugation and stained with CD11c-FITC, CD45-APC and Siglec-F-PE (BD Biosciences). BAL eosinophil levels were evaluated using a FACS Calibur (BD Biosciences) and flow cytometric data were analyzed by using FlowJo software.

### 2.8. ELISA

Levels of Abs in the immune sera were measured by enzyme-linked immunosorbent assay (ELISA). In brief, 96-well plates (Nunc, Roskilde, Denmark) were pre-coated with 100 µl/well of 2×10^3^ PFU of purified RSV A2 virus in PBS overnight at 4°C. After blocking with PBS containing 5% skim milk for 1 hr at room temperature, each serum sample was serially diluted in blocking buffer to each well and incubated for 1 hr at 37°C, followed by addition of 1∶3,000 diluted HRP-conjugated goat anti-mouse IgG (Southern Biotechnology, Birmingham, AL, USA). After incubation for 1 hr at room temperature, tetramethylbenzidine (TMB, Millipore) was added to develop the color and then the reaction was stopped by adding 2M H_2_SO_4_. The absorbance at wavelength 450 nm was measured by an ELISA reader (Molecular Devices, Sunnyvale, CA, USA). The endpoint titer was determined by O.D. cut-off values of 0.2.

### 2.9. Virus Titration in Mouse Lung

To determine the viral titers after challenge, lungs from RSV-infected mice were isolated on day 4 post-RSV challenge. The lungs were then strained through 70-µm cell strainer (BD Biosciences) using serum-free MEM. Supernatant was collected and RSV titers were determined by plaque assay using HEp-2 cells. The virus titer in the lung data are expressed as PFU per lung.

### 2.10. Statistical Analysis

Statistical differences were performed using GraphPad software. Data were analyzed for significance using one-way ANOVA with Bonferroni post-hoc test for multiple comparisons. The difference was considered statistically significant when the *P* value was less than 0.05.

## Results

### 3.1. Effect of CD4^+^ T Cell Epitope in wtAGcf on the Immune Response and Pathogenesis following RSV A Subtype Infection

**Figure 2:**
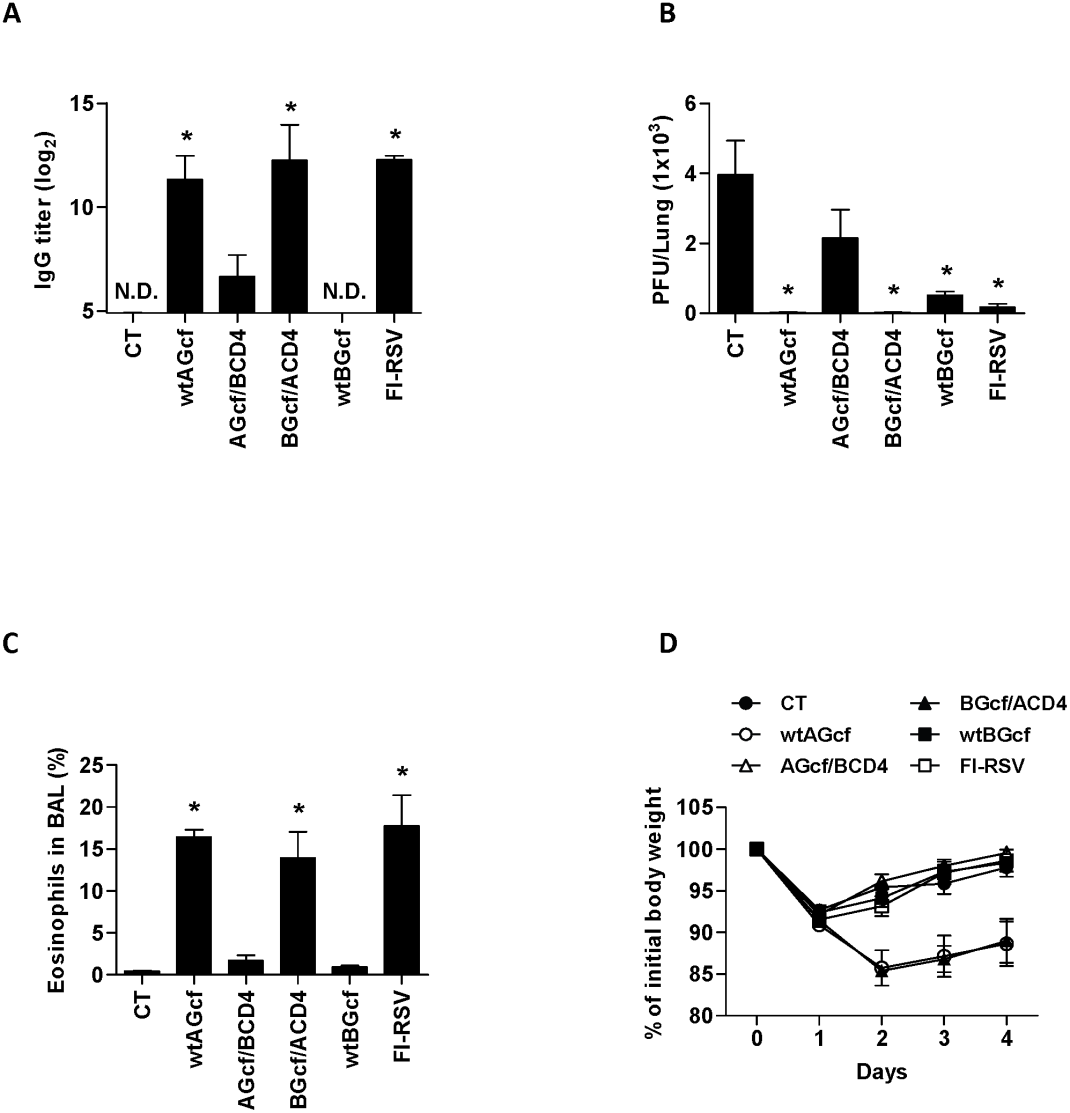
Effect of CD4^+^ T cell epitope on immune response and pathogenesis induced by wtAGcf. Mice were immunized twice with 20 µg of wtAGcf, AGcf/BCD4, BGcf/ACD4, or wtBGcf in the presence of 2 µg of CT or CT alone sublingually or with 1×10^5^ PFU of FI-RSV through foot-pad route. (A) RSV A2 specific serum IgGs were measured by ELISA 13 days after the second immunization. At day 4 post-challenge with 2×10^6^ PFU of RSV A2, (B) the viral replication in the lungs was determined by plaque assay and (C) the pulmonary eosinophils were measured and by flow cytometry. (D) Body weight loss was monitored daily after the viral challenge. The results are expressed as mean + S.E.M. for the group (n = 5). The data are representative of three separate experiments. Significant differences from results with the CT are *, *P*<0.05.

Neutralizing Ab is a major correlate of protection against RSV infection, and RSV G protein is known to induce neutralizing Ab responses in numerous studies [3]. Interestingly, however, previous study also demonstrated that adoptive transfer of RSVG-specific Type 2 CD4^+^ helper T cells from RSVG-sensitized mice causes severe pulmonary disease in recipient mice following RSV challenge [12]. Therefore, we examined whether immunization of mice with recombinantly modified Gcf, deficient in CD4^+^ T cell epitope, could offer protection against RSV infection while eliminating RSVG-mediated lung immunopathology. We immunized mice with the recombinant wild type Gcf derived from RSV A2 (wtAGcf), recombinant RSV A2 Gcf with its CD4^+^ T cell epitope replaced with the corresponding a.a. sequence from RSV B Gcf (AGcf/BCD4), recombinant RSV B Gcf with its CD4^+^ T cell epitope replaced with the corresponding a.a. sequence from RSV A2 Gcf (BGcf/ACD4), recombinant wild type Gcf derived from RSV B1 (wtBGcf), FI-RSV, or CT adjuvant alone. Subsequently, the levels of antigen-specific serum IgGs were measured 13 days after the second immunization. Our results showed that, compared to CT immunization, wtAGcf, BGcf/ACD4, and FI-RSV immunization induced significant levels of RSV A2-specific serum IgGs (Figure 2A) while wtAGcf, and BGcf/ACD4 immunization induced significant levels of wtAGcf-specific serum IgGs (Figure S1A). However, AGcf/BCD4 or wtBGcf immunization failed to induce significant RSV A2 or wtAGcf-specific serum IgG response.

Next, we examined virus titers in the lung, eosinophil infiltration in BAL, and body weight changes in immunized mice following RSV A2 challenge. At day 4 post-challenge, we detected no RSV in the lungs of mice immunized with wtAGcf or BGcf/ACD4. RSV titer in the lung was significantly decreased in mice immunized with wtBGcf or FI-RSV compared to control group immunized with CT alone. However, although a moderate reduction of viral titers in the lung was observed in mice immunized with AGcf/BCD4, this reduction was not significant (Figure 2B). Interestingly, we observed significant increase in the percent of eosinophils in BAL collected from mice immunized with wtAGcf, BGcf/ACD4, or FI-RSV compared to mice immunized with CT alone. However, AGcf/BCD4 or wtBGcf immunization did not cause such increase in the percentage of eosinophils in BAL (Figure 2C). Moreover, mice immunized with wtAGcf or BGcf/ACD4 experienced more severe weight loss and slower weight recovery following RSV challenge compared to other immunization groups (Figure 2D).

Our results indicate that CD4^+^ T cell epitope within RSV AGcf is essential for the induction RSV-specific Ab responses and viral clearance. However, these results also suggest that the same CD4^+^ T cell epitope is functionally linked to the enhancement of RSV disease as shown by significant increase in the eosinophilic recruitment to the airway mucosa and severe body weight loss only when the mice were immunized with Gcf containing this CD4^+^ T cell epitope. Taken together, our data suggest that CD4^+^ T cell epitope within RSV A2 Gcf possesses a dual function in mediating the development of protective immunity and vaccine-enhanced disease.

### 3.2. Immune Response and Eosinophilia by wtBGcf after RSV B Subtype Infection

**Figure 3:**
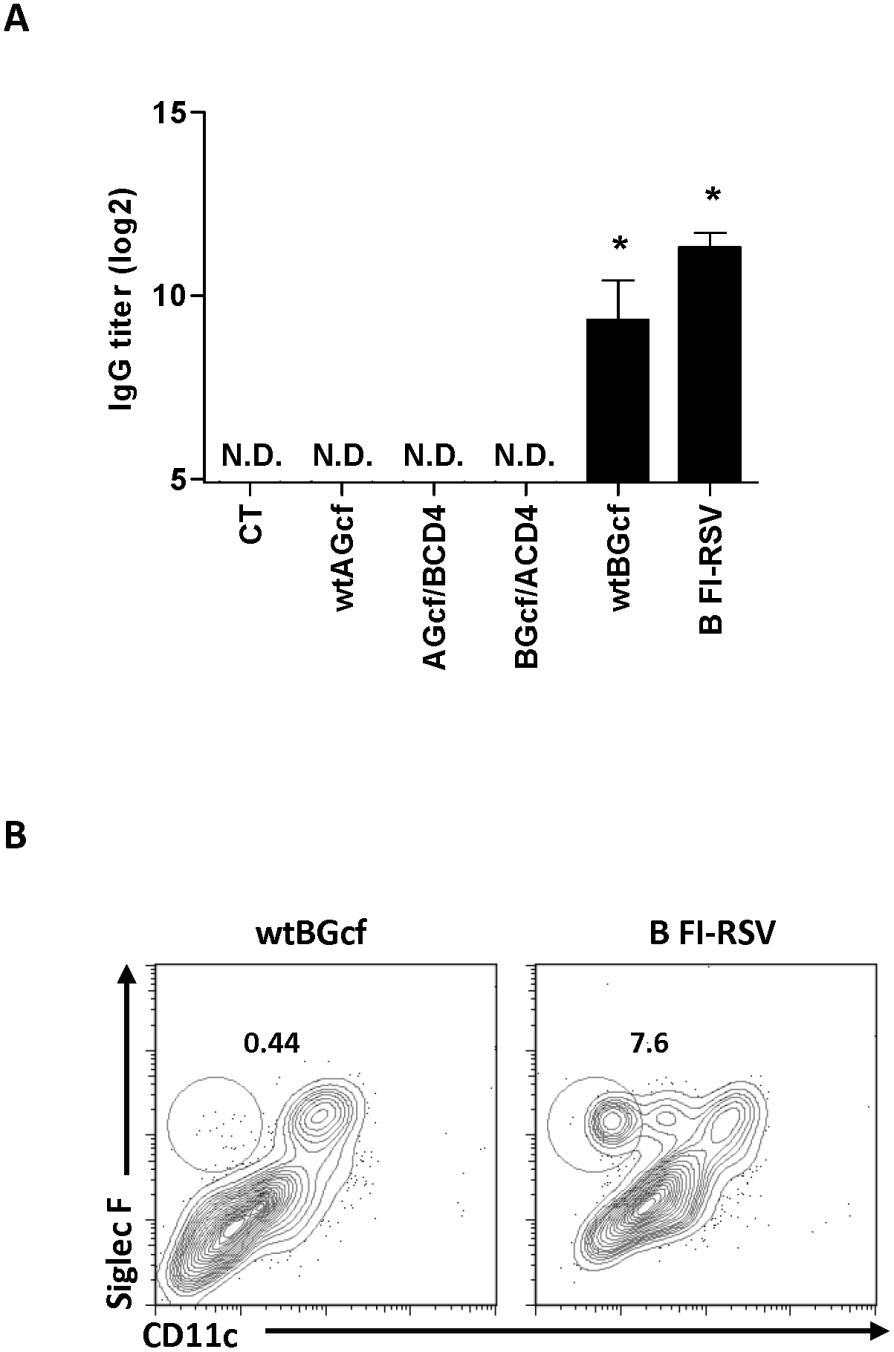
Antibody production and eosinophilia in mice immunized with wtBGcf. Mice were immunized twice with 20 µg of wtAGcf, AGcf/BCD4, BGcf/ACD4, or wtBGcf in the presence of 2 µg of CT or CT alone sublingually or with 1×10^5^ PFU of B type FI-RSV through foot-pad route. (A) RSV B type specific serum IgGs were measured by ELISA 13 days after the second immunization. (B) For the pulmonary eosinophilia, mice were immunized twice with 20 µg of wtBGcf in the presence of 2 µg of CT sublingually or with 1×10^5^ PFU of B type FI-RSV through foot-pad route. At day 4 post challenge with 2×10^6^ PFU of CH18537, the pulmonary eosinophils were detected by flow cytometry. The results are expressed as mean + S.E.M. for the group (n = 3). The data are representative of three separate experiments. Significant differences from results with the CT are *, *P*<0.05.

To examine whether RSV B Gcf shares the same region a.a. as a CD4^+^ T cell epitope, we immunized mice with wtAGcf, AGcf/BCD4, BGcf/ACD4, wtBGcf, FI-RSV derived from B subtype (FI-RSVB), or CT alone using the same immunization regiment used above and the levels of antigen-specific serum IgGs were measured 13 days after the second immunization. The results indicate that mice immunized with wtBGcf or FI-RSVB produced significantly (*P*<0.05) higher levels of RSV B-specific serum IgGs than those immunized with CT alone (Figure 3A). Although, the level of RSV B-specific serum IgGs generated by BGcf/ACD4 immunization was considerably low compared to that by wtBGcf or FI-RSVB immunization, it was noting that BGcf/ACD4 immunization induced significantly (*P*<0.05) higher levels of RSV B-specific serum IgGs than CT immunization, which was below the y-axis cut-off threshold of log (base 2) 5 (data not shown). Meanwhile, wtBGcf-specific serum IgGs were also induced in mice immunized with BGcf/ACD4 or wtBGcf (Figure S2B). It was worth noting that minimal levels of RSV B or wtBGcf-specific serum IgGs were detected in mice immunized with wtAGcf, AGcf/BCD4, or CT alone (Figure 3A).

Next, to examine whether immunization with wtBGcf could induce pulmonary eosinophilia following subsequent infection with RSV B subtype, we immunized mice twice with wtBGcf or FI-RSVB and challenged with RSV B subtype (CH18537) 14 days after the second immunization. At day 4 post-challenge, we observed considerable increase in the percentage of eosinophils in BAL harvested from mice immunized with FI-RSVB as expected, but not from wtBGcf-immunized mice (Figure 3B), indicating that immunization with wtBGcf, unlike wtAGcf, does not cause pulmonary eosinophilia upon subsequent infection with the homologous RSV subtype.

Taken together, these results demonstrate that immunization with RSV B Gcf induces RSV B–specific Ab responses without promoting excessive eosinophilic recruitment into the airway upon RSV B subtype challenge. Furthermore, a.a. 183–195 within RSV B Gcf is neither essential in the induction of RSV B-specific Ab responses and nor function as a CD4^+^ T cell epitope.

### 3.3. Amino Acid Residues 183–195 within RSV BGcf Lack Functionality as CD4^+^ T Cell Epitope

**Figure 4:**
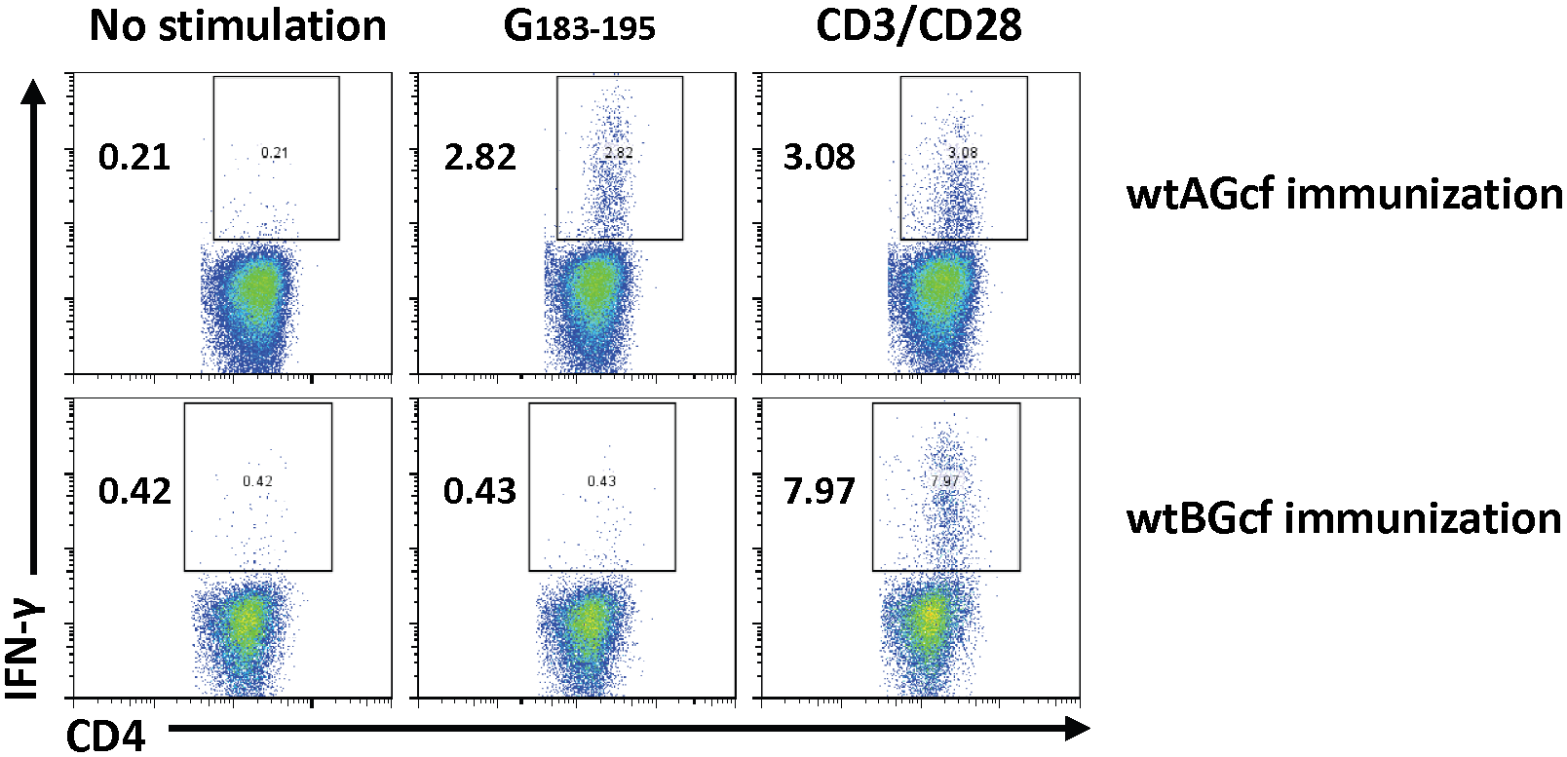
CD4 epitope in wtGcf. Mice were immunized twice with 20 µg of wtAGcf, or wtBGcf in the presence of 2 µg of CT sublingually in a prime-boost regimen. At day 4 post challenge with 2×10^6^ PFU of RSV A or B subtype, respectively, lung mononuclear cells were prepared from the lung tissue and re-stimulated with G peptides (a.a. 183–195) from wtAGcf or wtBGcf or 1 µg/ml of anti-CD3 and anti-CD28 antibodies as positive control. T cell responses were measured IFN-γ secreting CD4^+^ T cells by intracellular staining. The data are representative of three separate experiments.

We further validated the possibility that a.a. residues 183–195 within RSV BGcf contain CD4^+^ T cell epitope functionality. To this end, we immunized mice with either wtAGcf or wtBGcf in a prime-boost regimen and challenged them with RSV A or B subtype, respectively. At 4 day post-challenge, lung mononuclear cells were isolated and stimulated with wtBG_183–195_, wtAG_183–195_, or a mixture of anti-CD3 and -CD28 antibodies as positive control, and RSVG-specific IFN-γ response in the CD4^+^ T cells was evaluated. The results showed a robust induction of RSVG-specific IFN-γ response in CD4^+^ T cells in the lung mononuclear cells harvested from mice immunized with wtAGcf, but not from mice immunized with wtBGcf (Figure 4). Overall, these results confirm our hypothesis that a.a. residues 183–195 within RSV BGcf do not function as a CD4^+^ T cell epitope.

### 3.4. T Cell Response by Gcfs

A previous study by Varga *et al.* has shown that alanine-substitution at a.a. position 185 or 188 within RSVG can inhibit CD4^+^ T cell activation by RSVG [25]. Therefore, we generated a recombinantly modified Gcf (mGcf) in which two amino acids corresponding to a.a. positions 185 and 188 in Gcf were substituted with alanine by point-mutation. Furthermore, since eliminating the CD4^+^ T cell epitope would negate the induction of RSVG-specific immunity, another modified Gcf (Th-mGcf) was generated by fusing a CD4^+^ T cell epitope from RSV F protein (F_51–66_) to the N-terminus of mGcf to maintain CD4^+^ T cell-mediated RSVG-specific immune responses.

**Figure 5:**
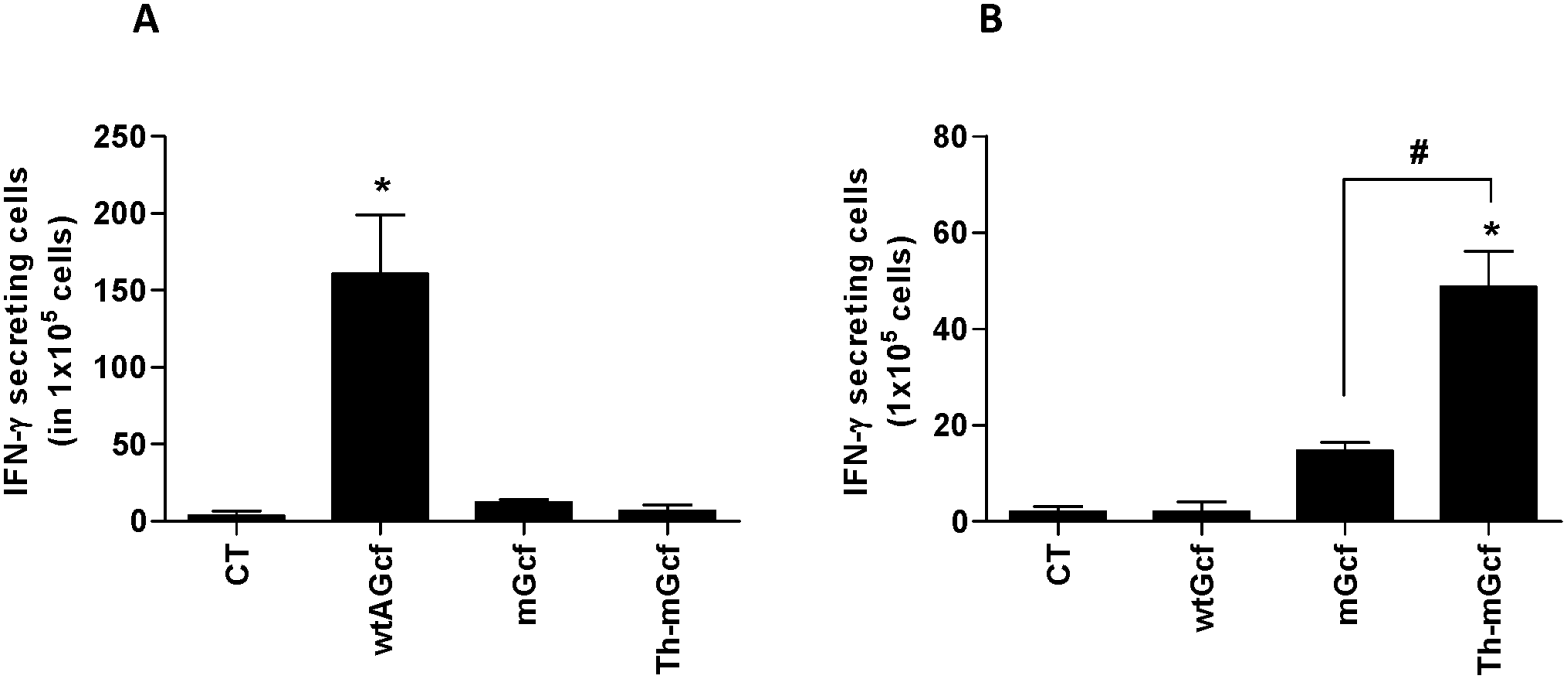
T cell response by modified Gcf. Mice was immunized twice with Gcfs sublingually or FI-RSV through foot-pad route and then challenged with RSV A2 three weeks after the second immunization. At day 4 post-challenge, lung mononuclear cells were prepared from the lung tissue and re-stimulated with (A) G (a.a. 183–195) or (B) F (a.a. 51–66) peptide. T cell responses were detected as IFN-γ secreting cells by ELISPOT. The results are expressed as means + S.E.M. for the group (n = 3). The data are representative of two separate experiments. Significant differences from results with the CT are marked with asterisks (*), while significant differences between mGcf and Th-mGcf are marked with sharp (#), respectively (* or #, P<0.05).

In order to examine the ability of modified Gcf to stimulate CD4^+^ T cells, mice were immunized sublingually in a prime-boost regimen with wtAGcf, mGcf, Th-mGcf, or CT and challenged with RSV A2. At day 4 post-challenge, lung mononuclear cells were isolated, stimulated with G_183–195_-(Figure 5A) or F_51–66_-(Figure 5B) and IFN-γ^+^ response was evaluated by ELISPOT assay. As expected, the numbers of IFN-γ-secreting cells were significantly (*P<0.05*) increased with G_183–195_ stimulation in cells from mice immunized with wtAGcf and with F_51–66_ stimulation in cells from mice immunized with Th-mGcf, respectively. We have further confirmed this by intracellular cytokine staining using flow cytometry (data not shown). Collectively, our results demonstrate that recombinantly fusing CD4^+^ T cell epitope from RSV F protein to mGcf, which lost its original G protein CD4^+^ T cell-stimulating functionality due to mutations within its T cell epitope, effectively restores mGcf’s ability to stimulated CD4^+^ T cells.

### 3.5 Immunogenicity and Protective Efficacy of Recombinant Gcfs against Homologous RSV A type Infection

**Figure 6:**
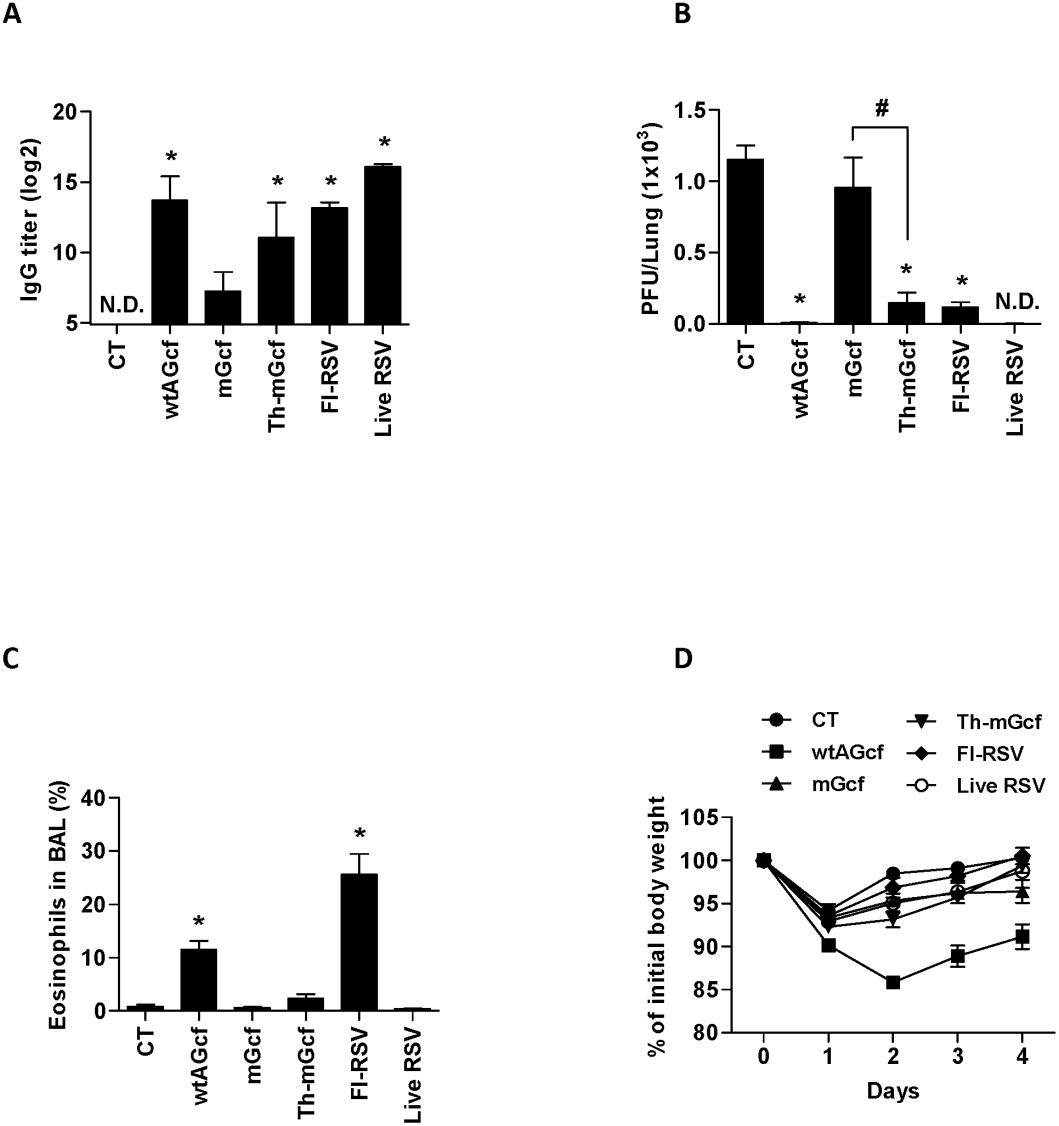
Effect of modified Gcf on the infection of homologous RSV A type. Mice (n = 5) were immunized with 20 µg of wtAGcf, mGcf, or Th-mGcf sublingually in the presence of 2 µg of CT, 1×10^5^ PFU of FI-RSV through foot-pad or 1×10^5^ PFU of live RSV A2 intranasally on days 0 and 14 and (A) RSV A2 specific serum IgG was measured by ELISA two weeks after the second immunization. The mice were challenged with 2×10^6^ PFU of RSV A2 three weeks after the last immunization. (B) The viral replication in the lungs was determined by plaque assay at day 4 post-challenge and (C) the pulmonary eosinophils were examined by flow cytometry. (D) The body weight loss was monitored daily after the viral challenge. N.D., not detected. The results are expressed as means + S.E.M. for the group (n = 5). The data are representative of three separate experiments. Significant differences from results with the CT are marked with asterisks (*), while significant differences between mGcf and Th-mGcf are marked with sharp (#) respectively (* or #, P<0.05).

Next, we examined various aspects of RSV-specific immune responses after wtAGcf, mGcf, or Th-mGcf immunization and compared with live RSV and FI-RSV immunization followed by RSV challenge. Mice were immunized with the respective antigen in a prime-boost regimen and, at 13 days after the second immunization, RSV A2 (Figure 6A) or wtAGcf (Figure S2)-specific serum IgG levels were determined by ELISA. The results demonstrate that mice immunized with wtAGcf, Th-mGcf, FI-RSV, and live RSV produced significantly (*P<0.05*) higher RSV A2-specific serum IgGs than the CT-immunized negative control (Figure 6A). In addition, immunization with wtAGcf, and Th-mGcf induced significantly (P<0.05) higher wtAGcf-specific serum IgGs than the negative control (Figure S2). Importantly, the level of RSV A2-specific serum IgGs in Th-mGcf immunized mice was greater than that in mGcf-immunized group. However, RSV A2-specific serum IgGs induced by Th-mGcf immunization were lower than those induced by wtAGcf, FI-RSV, or live-RSV immunization (Figure 6A).

In order to examine the efficacy of Th-mGcf immunization in facilitating viral clearance, immunized mice were challenged intranasally with RSV A2. At day 4 post-challenge, lungs were harvested from RSV-challenged mice, and viral plaque assays were performed with the lung homogenates to detect RSV titers. The results indicate that wtAGcf, Th-mGcf, and FI-RSV immunization significantly reduced lung viral titers compared to CT immunization, whereas mGcf immunization did not lead to a significant reduction of viral titers (Figure 6B). Notably, Th-mGcf immunization showed significantly enhanced lung viral clearance compared to mGcf immunization.

Furthermore, we examined eosinophil recruitment to BAL following RSV A2 challenge in Th-mGcf immunized mice as previous studies have shown that mice primed with RSVG experience exacerbated disease and pulmonary eosinophilia when subsequently challenged with RSV [26]. As expected, RSV challenge in FI-RSV immunized mice caused substantial recruitment of eosinophils, composing ∼25% of the BAL cells (Figure 6C). Mice that received wtAGcf immunization, although in lesser degree than FI-RSV immunization, also showed significant eosinophil recruitment following RSV challenge, composing ∼10% of the BAL cells. It is important to note that Th-mGcf immunization, along with mGcf or live RSV immunization, did not induce significant eosinophil recruitment. In addition, weight loss in immunized mice was measured following RSV challenge in order to assess the protection from morbidity conferred by Th-mGcf immunization. The results show that, while mice immunized with wtAGcf experienced ∼15% reduction in their body weight by day 2 post-challenge, weight loss in mice immunized with CT, mGcf, Th-mGcf, FI-RSV, or live RSV was less severe and began to recover their body weight at day 1 post-challenge (Figure 6D).

Taken together, these results indicate that fusing F_51–66_ to mGcf restores the ability of mGcf to induce RSV-specific Ab response and viral clearance. Furthermore, Th-mGcf did not induce excessive pulmonary eosinophil recruitment and body weight loss upon Gcf immunization followed by RSV challenge.

### 3.6 Immunogenicity and Protective Efficacy of Recombinant Gcfs against Heterologous RSV B type Infection

**Figure 7:**
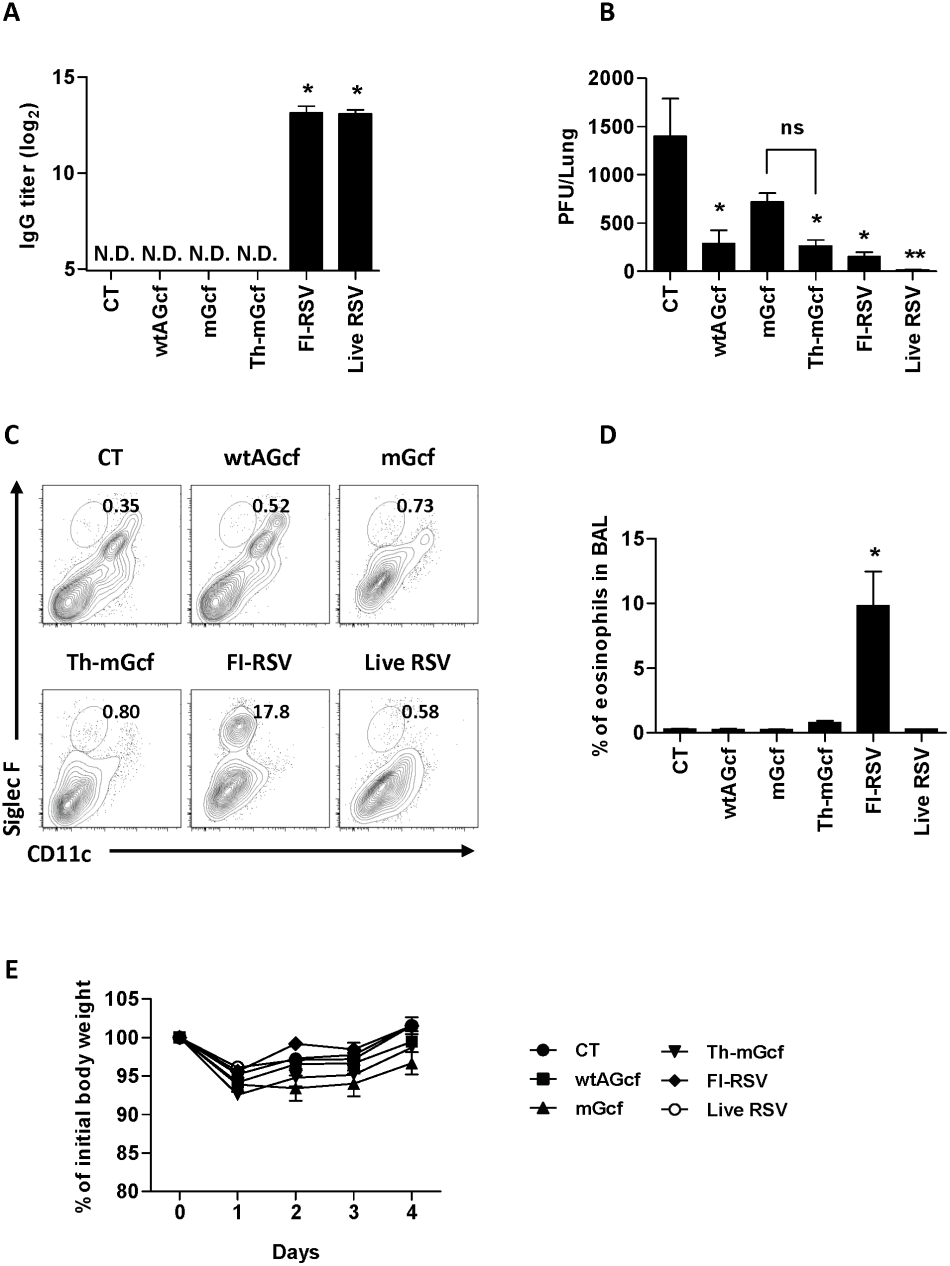
Effect of modified Gcf on the infection of heterologous RSV B type. (A) The mice (n = 5) were immunized with 20 µg of Gcf, mGcf or Th-mGcf sublingually in the presence of 2 µg of CT, 1×10^5^ PFU of FI-RSV through foot-pad or 1×10^5^ PFU of live RSV intranasally on days 0 and 14 and the level of RSV B type specific serum IgG was measured by ELISA 13 days after the second immunization. (B–D) The mice were challenged with 4×10^6^ PFU of RSV B type (KR/B/10–12) three weeks after the last immunization. (B) The level of viral replication in the lungs was determined by plaque assay at day 4 post-challenge and (C) the pulmonary eosinophils were examined by flow cytometry. (D) The body weight loss was monitored daily after the viral challenge. N.D., not detected. The results are expressed as means + S.E.M. for the group (n = 5). The data are representative of three separate experiments. Significant differences from results with the CT are marked with asterisks (*), while significant differences between mGcf and Th-mGcf are marked with sharp (#) respectively (* or #P<0.05).

RSV is classified into two different subtypes: A and B, depending on the a.a. sequence in its G protein [27]. In order to examine whether immunization with Th-mGcf, derived from RSV A subtype, can induce specific immune responses against the B subtype, mice were immunized with wtAGcf, mGcf, Th-mGcf, FI-RSV, live RSV A2, or CT alone, and RSV B-specific serum IgGs were measured 13 days after the last immunization (Figure 7A). Our results show that mice immunized with FI-RSV or live RSV A2 produced RSV B subtype-specific IgGs despite the use of RSV A subtype origin FI-RSV and live RSV for the immunization. However, mice immunized with wtAGcfs, mGcf, or Th-mGcf did not produce RSV B subtype-specific serum IgGs.

Next, immunized mice were challenged with RSV B (KR/B/10–12) subtype, and virus titer in the lung and eosinophil infiltration in BAL were compared at day 4 post-challenge. Our results indicate that, compared to CT immunization, immunization with wtAGcf, Th-mGcf, FI-RSV, or live RSV A2 led to a significant reduction in virus titer in the lung following RSV B (KR/B/10–12) subtype challenge (Figure 7B). The mGcf immunization also caused a slight decrease in the virus titer with no statistical significant. Such reduction in lung virus titers in wtAGcf and Th-mGcf immunized mice even in the absence of robust RSV B-specific IgG response may be attributed to G183–195- and F51–66-specific IFN-γ responses respectively. Moreover, significant increase in the percentage of eosinophils in BAL was observed in FI-RSV immunized mice as expected. However, immunization with wtAGcf, mGcf, Th-mGcf, or live RSV A2 showed few or no eosinophils in BAL (Figure 7C & D). Furthermore, no significant differences in weight loss were found among the immunization groups following RSV B subtype challenge (Figure 7E). Collectively, these results demonstrate that RSV A2-derived Th-mGcf can confer cross-protective immunity against both subtypes of RSV without causing vaccine-mediated enhancement of disease upon RSV infection.

## Discussion

RSV is a major respiratory pathogen in infants, elderly, and immunocompromised, often causing a severe lower respiratory tract infection. Currently, however, there is no licensed vaccine available. RSV G protein can be a promising RSV vaccine candidate because it induces RSV-specific antibody responses, although CD4^+^ T cell epitope within G protein has been shown to be related to immunopathology. In this regard, we engineered various recombinant RSV Gcfs which is RSV G protein fragment (a.a. 131–230), expecting protective immune response without immunopathology.

The results from the present study highlights: (1) unlike RSV A subtype, a.a. residues 183–195 in Gcf of RSV B subtype do not function as CD4^+^ T cell epitope, (2) Gcfs from RSV B subtype, unlike RSV A subtype, do not induce eosinophilia after RSV B subtype challenge, and (3) Gcf derived from RSV A subtype containing modified CD4^+^ T cell epitope can protect mice against both RSV A and B subtypes without immunopathology.

Studies have indicated that RSV A subtype infection occurs with higher frequency and may exhibit more severe disease symptoms than those of RSV B subtype infection, but both strains often circulate together during RSV epidemics and contribute to the RSV antigenic heterogeneity and reinfection [28], [29]. Moreover, shifting in the predominance of circulating RSV subtypes occurs in 1–2 year cycle [30]. Therefore, an effective RSV vaccine should confer protective immunity against both subtypes of RSV. However, there has been about very limited studies on the vaccine against RSV B subtype infection.

It is well known that G protein is antigenically variable and the sequence homology of G proteins between A and B subtype is approximately 53% [31]. Nevertheless, G protein of RSV B subtype also contain 13 a.a. peptide which is conserved in both subtypes of RSV [32], 4 conserved Cys residues [33], and a CX3C chemokine motif [34]. We investigated whether wtBGcf containing these regions can induce protection against RSV infection. Both wtAGcf and wtBGcf elicit RSV-specific serum IgG against the homologous subtype, but not against the heterologous subtype. Importantly, wtBGcf did not induce eosinophilia and a.a. 183–195 in wtBGcf was identified to not possess CD4^+^ T cell epitope functionality in contrast to wtAGcf, which has a dual function of eliciting RSVG-specific CD4^+^ T cell immunity and promoting immunopathology. Our study indicatess that wtBGcf does not induce vaccine-enhanced disease. However, our results suggest that CD4^+^ T cell epitope in wtBGcf may be present at a different region other than a.a. 183–195, because the mice immunized with wtBGcfs produced RSV-specific serum IgGs, suggesting helper T cell activity must have fostered B cell class switching.

Numerous studies demonstrated that G protein is critically associated with immunopathology in mice immunized with G protein followed by RSV infection. It has been shown that the mice immunized with conserved region of RSV G protein reduced the various parameters of RSV disease, including weight loss, pulmonary inflammation, and lung viral titer [35], [36]. Importantly, however, RSV G protein appears to be involved in the enhancement of RSV disease pathogenesis in RSVG-primed animals by eliciting aberrant T helper cell responses [2], [12], [17], [37]. It has been reported that a.a. residue 184–193 of RSV G protein is associated with pulmonary eosinophilia [18]. Accordingly, we have also shown in this study that CD4^+^ T cell epitope within RSV A2 Gcf is directly correlated, not only with the induction of RSVG-specific antibody responses and viral clearance, but also with the vaccine-mediated eosinophilia and severe body weight loss.

In the previous study, we demonstrated that a region within the RSV Gcf containing four cysteine residues (CX3C motif) is responsible for the induction of RSVG-specific immune response [22]. In order to maintain the immunogenicity while eliminate CD4^+^ T cell epitope-mediated eosinophilia, mGcf which lost the function of CD4^+^ T cell epitope [25] and Th-mGcf was generated. This new Th-mGcf induced RSV-specific IgG response and viral clearance effect, but not pathogenesis. In addition, mice immunized with Th-mGcf had increased number of F_51–66_–specific IFN-γ-secreting cells in the lung, suggesting that Th-mGcf immunization may also boost the aspects of cell-mediated immunity that are important for viral clearance (eg. Th1 or CTL response).

Interestingly, mice immunized with Th-mGcf did not exhibit strong RSV B subtype specific serum IgG response, but significantly enhanced viral clearance without causing severe weight loss or exaggerated recruitment of eosinophil to the airway mucosa. Given the light of the fact that mice immunized with mGcf, without the F_51–66_ peptide fusion, failed to effectively clear the virus, it is probably that the protection from RSV B subtype was rendered by Th-mGcf’s ability to generate F_51–66_ –specific IFN-γ response.

In conclusion, our findings describe that the vaccination approach using the modified Gcf engineered to eliminate its original T cell epitope may be a promising strategy in developing a novel RSV vaccine for cross-protection against the both subtypes of RSV while preventing RSVG-mediated lung immunopathology.

## Supporting Information

Figure S1 Protein-specific antibody in mice immunized with wtAGcf, AGcf/BCD4, BGcf/ACD4, wtBGcf, and FI-RSV. Mice were immunized twice with 20 µg of wtAGcf, AGcf/BCD4, BGcf/ACD4, or wtBGcf in the presence of CT, CT alone sublingually or 1×10^5^ PFU of FI-RSV through foot-pad route. (A) wtAGcf or (B) wtBGcf specific serum IgG were measured by ELISA 13 days after the second immunization. The results are expressed as means + S.E.M. for the group (n = 5). The data are representative of three separate experiments. Significant differences from results with the CT are marked with asterisks (*, P<0.05).
(TIF)

Figure S2 wtAGcf-specific antibody in mice immunized with wtAGcf, mGcf, Th-mGcf, FI-RSV and live RSV. Mice were immunized twice with wtAGcf, mGcf, or Th-mGcf in the presence of CT, CT alone sublingually, 1×10^5^ PFU of FI-RSV through foot-pad route or 1×10^5^ PFU of live RSV A2 intranasally. wtAGcf specific serum IgG were measured by ELISA 13 days after the second immunization. The results are expressed as means + S.E.M. for the group (n = 5). The data are representative of three separate experiments. Significant differences from results with the CT are marked with asterisks (*, P<0.05).
(TIF)
